# Functional recovery after accidental deep hypothermic cardiac arrest: Comparison of different cardiopulmonary bypass rewarming strategies

**DOI:** 10.3389/fphys.2022.960652

**Published:** 2022-09-05

**Authors:** Ole Magnus Filseth, Timofei Kondratiev, Gary C. Sieck, Torkjel Tveita

**Affiliations:** ^1^ Anesthesia and Critical Care Research Group, Faculty of Health Sciences, Department of Clinical Medicine, UiT The Arctic University of Norway, Tromsø, Norway; ^2^ Division of Surgical Medicine and Intensive Care, University Hospital of North Norway, Tromsø, Norway; ^3^ Emergency Medical Services, University Hospital of North Norway, Tromsø, Norway; ^4^ Department of Physiology and Biomedical Engineering, Mayo Clinic, Rochester, MN, United States

**Keywords:** hypothermia induced, hypothermia accidental, cardiopulmonary bypass, immersion cooling, rewarming, cardiac index, cerebral microdialysis, cerebral blood flow

## Abstract

**Introduction:** Using a porcine model of accidental immersion hypothermia and hypothermic cardiac arrest (HCA), the aim of the present study was to compare effects of different rewarming strategies on CPB on need for vascular fluid supply, level of cardiac restitution, and cerebral metabolism and pressures.

**Materials and Methods:** Totally sixteen healthy, anesthetized castrated male pigs were immersion cooled to 20°C to induce HCA, maintained for 75 min and then randomized into two groups: 1) animals receiving CPB rewarming to 30°C followed by immersion rewarming to 36°C (CPB_30_, *n* = 8), or 2) animals receiving CPB rewarming to 36°C (CPB_36_, *n* = 8). Measurements of cerebral metabolism were collected using a microdialysis catheter. After rewarming to 36°C, surviving animals in both groups were further warmed by immersion to 38°C and observed for 2 h.

**Results:** Survival rate at 2 h after rewarming was 5 out of 8 animals in the CPB_30_ group, and 8 out of 8 in the CPB_36_ group. All surviving animals displayed significant acute cardiac dysfunction irrespective of rewarming method. Differences between groups in CPB exposure time or rewarming rate created no differences in need for vascular volume supply, in variables of cerebral metabolism, or in cerebral pressures and blood flow.

**Conclusion:** As 3 out of 8 animals did not survive weaning from CPB at 30°C, early weaning gave no advantages over weaning at 36°C. Further, in surviving animals, the results showed no differences between groups in the need for vascular volume replacement, nor any differences in cerebral blood flow or pressures. Most prominent, after weaning from CPB, was the existence of acute cardiac failure which was responsible for the inability to create an adequate perfusion irrespective of rewarming strategy.

## Introduction

Over the past 4–5 decades, overall mortality of accidental hypothermia patients has decreased from 52 to 80% (Maclean and Emslie-Smith, 1977; Murray and Hall, 1998) in previous reports to the present 28–35% (Roeggla et al., 1994; Megarbane et al., 2000; Vassal et al., 2001; van der Ploeg et al., 2010). This improved outcome has been closely linked to hypothermic patients with maintained spontaneous circulation during rescue and rewarming, whereas in patient with hypothermic cardiac arrest (HCA) the survival rate is much lower. However, case reports are documenting survival with favorable neurologic outcome after rewarming patients in HCA (Walpoth et al., 1997; Gilbert et al., 2000; Mark et al., 2012; Wanscher et al., 2012; Boue et al., 2014; Meyer et al., 2014) have raised optimism for creating new treatment algorithms to further increase survival also in this patient category. The focus in recent guidelines has been to improve the “chain of survival” by performing continuous cardio-pulmonary resuscitation (CPR) during transfer to a hospital where rewarming can be provided using extracorporeal rewarming. However, the overall mortality in patients with HCA admitted to two different university clinics in Norway has been estimated to be around 74% (Hilmo et al., 2014; Svendsen et al., 2017). Still, the potential for survival with good neurological outcome in hypothermic patients is now considered better than for normothermic patients with witnessed, unwitnessed, or asystolic cardiac arrest (Paal et al., 2022). In the subgroup of HCA patients where asphyxia did not precede cardiac arrest a survival rate of 46.7% was reported (Svendsen et al., 2017). Thus, it appears that improvements in the treatment of HCA patient may be able to lower mortality rate even further. Central aspects of the modified strategies appear related to rewarming methods and the rate of rewarming.

Rewarming methods used in accidental hypothermia patients have been mostly adapted from modern cardiac surgery, where cardio-pulmonary bypass (CPB) has been routinely applied over the past 60 years to cool and rewarm patients (Sealy et al., 1957). Although the overall morbidity and mortality associated with the use of CPB has steadily decreased since its introduction in cardiac surgery, hypothermic CPB can lead to post-hypothermic impairment of cardiac ventricular function (Schultz et al., 2006) and central nervous system complications (Newman et al., 1995; Selnes et al., 1999; Grigore et al., 2002; Newman et al., 2006; Grigore et al., 2009; Debaty et al., 2016). Hypothermic CPB invariably causes increased extravasation of fluid and general edema (Heltne et al., 2001; Farstad et al., 2003). The extravasation of plasma fluid takes place during normothermic CPB but increases significantly during hypothermic CPB (Heltne et al., 2001; Farstad et al., 2003). Case studies (Newman et al., 1995), as well as randomized controlled studies (Mora et al., 1996; Grigore et al., 2002) have shown that fast rewarming rates and inaccurate temperature management during the CPB period following cardiac surgery may lead to uncontrolled cerebral hyperthermia, which may potentially lead to worse neurologic and neurocognitive outcomes. Thus, current clinical recommendations simply call for avoiding hyperthermia during cardiac surgery by applying a slow rewarming rate; < 0.5°C/min, once temperature exceeds 30°C (Enomoto et al., 1996; Engelman et al., 2015). It is unclear whether these recommendations are also valid when using CPB to rewarm accidental hypothermia patients in HCA.

We recently observed significant differences in outcome in pigs cooled and rewarmed on CPB as used in cardiac surgery when compared to animals which were immersion cooled and rewarmed on CPB as in accidental hypothermia. Immersion cooled animals displayed significantly reduced survival due to severe cardiovascular failure after weaning from CPB. These animals also displayed signs of ischemic regional brain damage and a significantly greater need for vascular volume supply during CPB rewarming. This may indicate the existence of increased capillary fluid leak and extravasation during CPB rewarming in accidental hypothermia patients with potential impact on tissue edema. However, for most organs there is no definite association between tissue edema and organ function, nor organ survival. One exception is the brain, where tissue edema and elevated intra cranial pressure (ICP) may lead to cessation of cerebral circulation.

Aim of the present study was to test possible organ protective effects of different recommendations/guidelines (Paal et al., 2022), (Sahu et al., 2009) for rewarming accidental hypothermia patients in HCA, advocating for slow rewarming and early weaning from CPB. We tested effects of differing rewarming modalities on the need for vascular volume supply, the extent of tissue edema, changes in cerebral metabolism/blood flow and pressures, and in cardiovascular function after rewarming. To do this, pigs were immersion cooled to 20°C and maintained for 75 min in HCA. Thereafter, the animals were rewarmed at a similar rate from 20° to 30°C on CPB. Animals were then split into two groups: 1) rewarmed on CPB to 36°C, and 2) rewarmed to 36°C by water immersion. The rewarming rate on CPB from 30° to 36°C was more than two times faster than the group rewarmed by immersion.

## Material and methods

Castrated male pigs (*n* = 16, body wt. 24–39 kg) from a native Norwegian stock (norsk landsvin) were used. All animals received humane care in accordance with the Norwegian Animal Welfare Act. The study was approved by the Norwegian Animal Research Authority, (reference number: 08/03) and the animals were placed in pens for 2–5 days after arrival to the laboratory animal unit. They were fed twice daily and always had free access to water. After an overnight fasting, animals were cooled to 20°C by submersion in cold water, and after a 75-min period of HCA, they were randomly assigned to 1) rewarming by CPB to 30°C followed by immersion rewarming (CBP_30_; *n* = 8), or 2) rewarming by CPB to 36° (CPB_36_; *n* = 8).

### Anesthesia and hemodynamic monitoring

After an overnight fast, anesthesia was induced by an intramuscular bolus injection of a solution consisting of ketamine hydrochloride 20 mg/kg, midazolam 25 mg and atropine 1.0 mg. Peripheral venous catheters were then inserted into vein in either ear, and anesthesia was deepened by an intravenous bolus injection of fentanyl 10 µg/kg and thiopental 10 mg/kg. After tracheostomy a continuous infusion of fentanyl 20 µg/kg/h, thiopental 4 mg/kg/h and midazolam 0.3 mg/kg/h along with Ringer’s acetate 9 ml/kg/h was introduced via a 6 French (F) introducer side port catheter (Edwards Lifesciences, Irvine, CA, United States) placed in the right external jugular vein.

Intravenous anesthesia was maintained throughout the experiment, except during the period when brain temperature was below 25°C. After termination of the experiment, the anaesthetized animals were killed with an intravenous bolus injection of 20 mmol KCl. No neuromuscular blockers were used at any time. After tracheostomy, the animals were maintained on positive pressure ventilation (Siemens Servo 900, Sweden). Supplemental O_2_ was given to achieve a slightly supranormal value for P_a_O_2_. Alveolar ventilation was adjusted to achieve a P_a_CO_2_ of 4.5–6 kPa uncorrected for temperature (α-stat management). An arterial catheter was positioned into the left femoral artery for monitoring arterial pressure and for blood sampling. A 5F thermodilution catheter (Edwards Lifesciences) was placed through the introducer into the right external jugular vein for monitoring pulmonary arterial pressure, recording of blood temperature, measuring cardiac output (CO) and taking blood samples for determination of central venous saturation. A 6F pigtail high-volume-flush catheter (Cordis, Miami, FL, United States) was positioned into the left ventricle of the heart through the left carotid artery for monitoring of left ventricular pressures and administering fluorescent microspheres. A 14 F catheter was placed in the bladder through an extra-peritoneal incision for monitoring of urinary output throughout the experiment.

Electrocardiogram (ECG), heart rate (HR), central venous pressure (CVP), mean arterial pressure (MAP), left ventricular pressure and pulmonary arterial pressure (PAP) were continuously displayed on a 565A Patient Data Monitor (Kone, Espoo, Finland) and recorded at different time intervals by a special computer program developed by our department using the software package LabVIEW _TM_ v.6.0 (National Instruments, Austin, TX, United States). Cardiac output was measured by injecting 5 ml precooled saline in the thermodilution catheter positioned in the pulmonary artery. Thermodilution curves were displayed, and CO was computed on a Vigilance monitor (Edwards Lifesciences) and recorded manually.

### Brain microdialysis and intracerebral monitoring

An area of approximately 2 × 5 cm of the skull over the right hemisphere was exposed by excision of the scalp. A microdialysis catheter (CMA 70, CMA/Microdialysis, Stockholm, Sweden) was placed to a depth of 10 mm below the dura mater through a cranial hole 1 cm right of the sagittal suture and 2 cm rostral to the coronal suture. The catheter was connected to a 1.0 ml syringe placed into a microinfusion pump (CMA102, CMA/Microdialysis) and perfused with Ringer solution at a rate of 2.0 µl/min (Perfusion Fluid CNS, CMA/Microdialysis). Sampling time was 30 min at five times during the experiment: At baseline, at 30°C in the urinary bladder (bladder) during cooling, at HCA, at 30°C in the bladder during rewarming and after 2 h immersion rewarming to 38°C in the bladder. The microvials containing the dialysate fluid were immediately frozen at −70°C and concentrations of cerebral tissue glucose, lactate, pyruvate, glutamate, and glycerol were measured using a microdialysis analyzer (CMA 600, CMA/Microdialysis).

A pressure-monitoring catheter (Codman Micro-Sensor ICP Transducer, Codman & Shurtleff, Raynham, MA, United States/Millar transducer control unit TC-510, Houston, TX, United States) for recording of intracranial pressure, and a temperature probe for monitoring intracerebral temperature were placed in the brain parenchyma just below the dura mater through another cranial hole 1 cm to the right of the sagittal suture and 1 cm dorsal to the coronal suture. The intracranial pressure was displayed continuously on the Kone monitor and recorded manually at different time intervals.

A catheter was inserted in the jugular vein and advanced to the jugular bulb to sample blood for determination of brain venous O_2_ saturation.

### Injection and retrieval of fluorescent microspheres

We previously reported the detailed methods for blood flow measurements using this porcine animal model (Filseth et al., 2022). Briefly, a number of 1,000,000, 15 μM diameter fluorescent polystyrene microspheres (Molecular Probes, Eugene, OR, United States) were ultrasonicated, vortex-mixed and injected into the left ventricle (spontaneous circulation) or in the arterial line (cardiopulmonary bypass) four times in different colors (orange [excitation/emission wavelengths: 540/560 nm], yellow-green [505/515 nm], blue-green [430/465 nm], crimson [625/645 nm] and red [580/605 nm]) during the experiment: At baseline, during cooling at 30°C (bladder), during rewarming at 30°C (bladder), and at the end of experiment 2 h after rewarming to 36°C (bladder). At start of injection, blood was sampled at a rate of 4.12 ml/min for 2 min from the left femoral arterial catheter using a syringe mounted in a Harvard withdrawal pump for determination of reference blood flow. After each experiment, brain biopsies were obtained from the frontal lobes, cerebellum (2–3 g), and hippocampus (0.5–1 g), placed into sampling vials and later processed for determination of regional blood flow. For detection of fluorescence intensity, the tissue samples were ingested for 24 h at 60°C in sodium hydroxide. The microspheres were trapped in a sample filter unit (Germany) and dissolved in 98% 2-Ethoxyethyl acetate (Cellosolve) (Sigma-Aldrich Chemie, Steinheim, Germany). The fluorescent dyes were analyzed in a spectrofluorometer (Wallac Victor^2^TM 1420 Mulitlabel Counter, Perkin-Elmer Life Sciences, Turku, Finland). The fluorescence was compared to reference fluorescence in batches with dye from a known number of microspheres. Calculation of organ blood flow (OBF) was done according to the formula:

$$\mathrm{OBF}\;\left(\mathrm{ml}\;\cdot g^{\mathrm{-1}}\cdot \;\min^{-1}\right)=\left(R\;\cdot \;n_{T}\right)/n_{R}\cdot \mathrm{Wt}$$

R = withdrawal rate of the reference blood sample (4.12 ml/min); n_T_ = number of microspheres in the tissue sample; n_R_ = number of microspheres in the reference blood sample; Wt = weight of the tissue sample (g).

Alternatively:

$$\mathrm{OBF}\;\left(\mathrm{ml}\;\cdot \;g^{\mathrm{-1}}\;\cdot \;\min^{-1}\right)=\mathrm{(R}\;\cdot \;I_{T}\mathrm{)/}I_{R}\;\cdot \;\mathrm{Wt}$$

I_T_ = fluorescence intensity of the tissue sample; I_R_ = fluorescence intensity of the reference blood sample.

### Temperature monitoring

Body temperature was monitored continuously at four places: Intracerebral (Licox temperature probe/Licox MCB Universal oxygen and temperature monitor, Mielkendorf, Germany), esophagus (Kone temperature probe), bladder (Kone temperature probe), and blood in the pulmonary artery (5F thermodilution catheter, Edwards Lifesciences). During CBP, temperature was also monitored in the venous and arterial lines. Bladder temperature was used as reference temperature at 30°C during cooling and rewarming, and at 36°C during rewarming. Otherwise, esophageal temperature was used as reference temperature. Brain temperature was used to withdraw anesthetics at temperatures below 25°C.

### Experimental protocol

#### Immersion cooling

After instrumentation, all wounds were sutured in two layers and baseline measurements were obtained. Thereafter, a tub consisting of vinyl tarpaulin was rigged around the operating table and filled with cold water and ice slush. The animals were placed in a left- sided lateral position with 2/3 of the body submerged in cold water. The water temperature was kept at 5–10°C by circulation of the water through a heat-exchanger (Stöckert Normo/hypothermie, Munich, Germany). The upper right side of the animals was irrigated by cold water from the heat exchanger. The head was placed on a cushion to prevent contamination of the scalp wound and to protect the intracerebral monitoring. Spontaneous HCA occurred during immersion cooling at 20–19°C.

#### Cardiopulmonary bypass

After HCA, the tub was drained, and a right thoracotomy was performed in the fourth intercostal space. The right thoracic vessels were ligated, the pericardium was opened, and the heart and great vessels were exposed. A membrane oxygenator (Jostra Quadrox, Maquet Cardiopulmonary, Erlangen, Germany), a hosing system without venous reservoir and a centrifugal pump head (Bio-Medicus, Eden Prairie, MN, United States) were primed with approximately 600 ml of Ringer acetate. The CPB circuit was heparin coated. The ascending aorta was cannulated with a Jostra 16F arterial cannula, and the right atrial appendage was cannulated with a single 24F atrial cannula (Medtronic, Cardiac Surgical Products, Grand Rapids, MI, United States). A 12F intracardiac catheter was positioned into the left ventricle through the apex of the heart for decompression of the left ventricle until a sustainable cardiac rhythm was established. After 75 min of HCA, non-pulsatile CPB was initiated by a Bio-Medicus centrifugal pump. In the state of deep hypothermia, no heparin was administered, but blood coagulability was monitored using activated clotting time (ACT) (Hemochron 801, Hemochron whole blood coagulation system, Edison, NJ, United States). As rewarming proceeded, heparin was given to keep ACT around 200 s. During the initial 10 min, the flow rate was kept at 0.6 l/min and no temperature gradient was initiated. Thereafter, pump flow was adjusted to reach a global perfusion pressure above 50 mm Hg and maintain a central venous saturation above 60%. If these endpoints were not reached by increasing the pump head rotation speed, Ringer acetate was added to the circuit. Fresh gas administration was adjusted to keep P_a_O_2_ supranormal and to keep P_a_CO_2_ within normal limits according to the α-stat management. After the initial 10 min of CPB, rewarming was started by the Stöckert heat-exchanger, initiating a temperature gradient of 5°C between drained blood in the venous line and inflowing blood in the arterial line until the blood in the arterial line had reached the allowed maximum of 39°C. However, the temperature gradient between the bladder and blood in the inflowing arterial line was not allowed to exceed 10°C.

Internal electroconversion of ventricular fibrillation was initiated at an esophageal temperature of 25°C. If several attempts at electroconversion were unsuccessful up to an esophageal temperature of 28°C, 150 mg amiodarone was administered in the arterial cannula before additional attempts at electroconversion were made.

#### Weaning from cardio-pulmonary bypass

Animals in the CPB_30_ and the CPB_36_ groups were weaned from CPB at a bladder temperature of 30° and 36°C, respectively. Shortly before weaning, an intravenous dopamine infusion of 20 mg/h was started. The decision for applying dopamine was based on our previous experiment exploring cardiovascular effects of this drug at low temperatures (Filseth et al., 2012). The dopamine infusion rate was reduced or discontinued after weaning at MAP≥ 60 mm Hg. After cessation of CPB, blood in the hosing system and oxygenator was returned to the animals. The venous and arterial cannulas were removed, and the thoracotomy closed.

#### Rewarming after weaning from cardio-pulmonary bypass

After weaning at a bladder temperature of 30°C the animals in the CPB_30_ group were rewarmed to a bladder temperature at 36°C by immersion in warm water with a maximum temperature of 42°C. Animals in both groups were allowed to drift from a bladder temperature at 36°C to 38–39°C during 2 h by use of a warming mattress and a heating lamp.

#### Determination of gross weight before and after experiment and determination of dry/wet tissue weight

After the initial intramuscular bolus injection of anesthetic, the animals were weighed prior to the experiment. This initial weight was used for comparison to repeated weighing immediately after the cessation of the experiment.

The animals were killed 2 h after rewarming to 36°C (bladder), and 1–3 g tissue samples from brain, heart, lung, liver, kidney, gut, and muscle (long adducting muscle) were harvested and immediately put in liquid nitrogen. Later, the sample wet weight was determined (Mettler AE 163, Mettler-Toledo AG, Greifensee, Switzerland). After drying in incubator at 36°C for 24 h, the dry weight was determined, and dry/wet ratio was calculated.

#### Blood sample analysis

During the experiment, O_2_ and CO_2_ partial pressures, O_2_ saturation, pH, base excess, hemoglobin, sodium and potassium (Rapid lab 860, Ciba-Corning, Medfield, MA, United States) in systemic arterial blood, pulmonary arterial blood and internal jugular venous blood were recorded at: baseline, during cooling at 34°C (esophagus), 30°C (bladder), 25°C (esophagus) and during rewarming at the start of CPB, and at 25°C, 30°C, 34°C, and 36°C (all esophagus), at 30 and 36°C (bladder), and after 2 h rewarming to 36°C (bladder). During the experiment, measurements of blood glucose (HemoCue, Ängelholm, Sweden) leukocyte count, platelet count, red blood cell count, hemoglobin, and hematocrit (Cell analyzer CA 460, Medonic AB, Sweden) were recorded at baseline, during cooling at 30°C (bladder), shortly after initiation of CPB, during rewarming at 30°C (bladder), and at 2 h after rewarming to 36°C (bladder).

#### Statistical analysis

Statistical analyses were performed using Sigma Plot statistical software version 14 (Systat Software Inc. (SSI), Richmond, CA, United States). Normal distributions were assessed using the Shapiro-Wilk test. Differences between the groups for HR, SV, dry-to-wet weight ratio, weight gain ratio and fluid added during experiment were assessed using the Student *t*-test for normally distributed data or the Mann-Whitney rank sum test for non-normally distributed variables. Intragroup comparisons of data for HR and SV were performed by one-way repeated measures ANOVA for normally distributed variables, and Friedman repeated measures ANOVA on ranks for non-normally distributed variables. Where significant differences were found, Bonferroni *t*-test was used to compare values within group vs. baseline. All other data were assessed using two-way RM ANOVA. As a post hoc test for data of blood plasma metabolites, blood gases, blood cells count, CVP and ICP, Bonferroni *t*-test for multiple comparison was used to compare data both between and within groups, while for data of other hemodynamic values, oxygen transport, brain blood flow and brain metabolites Bonferroni *t*-test for comparison vs. control (CPB_30_ to compare data between groups and normothermic baseline to compare data within groups) was used. For comparison of survival between groups the two-sided Barnard’s unconditional test was used. The level of significance was set at *p* < 0.05. Data are presented as means and SD.

## Results

No statistically significant differences were found between groups in any variables during pre-hypothermic baseline measurements.

### Duration of cooling and rewarming, rewarming rates, and temperature measurements

**FIGURE 1:**
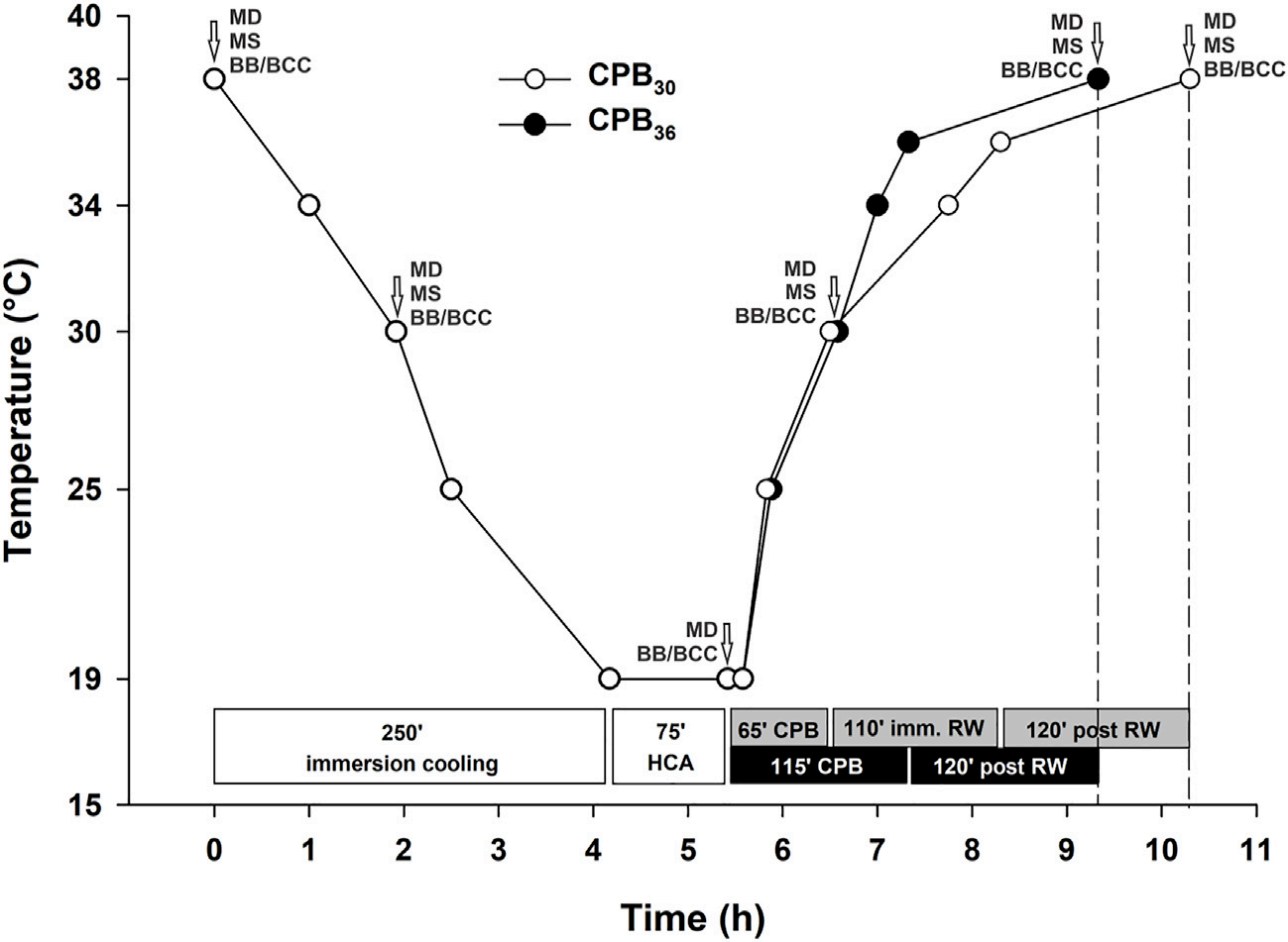
Experimental protocol. HCA – hypothermic cardiac arrest; CPB – cardiopulmonary bypass; RW – rewarming; imm. RW – immersion rewarming; MD – microdialysis; MS – microspheres; BB/BCC blood biochemistry/blood cells count.

**FIGURE 2:**
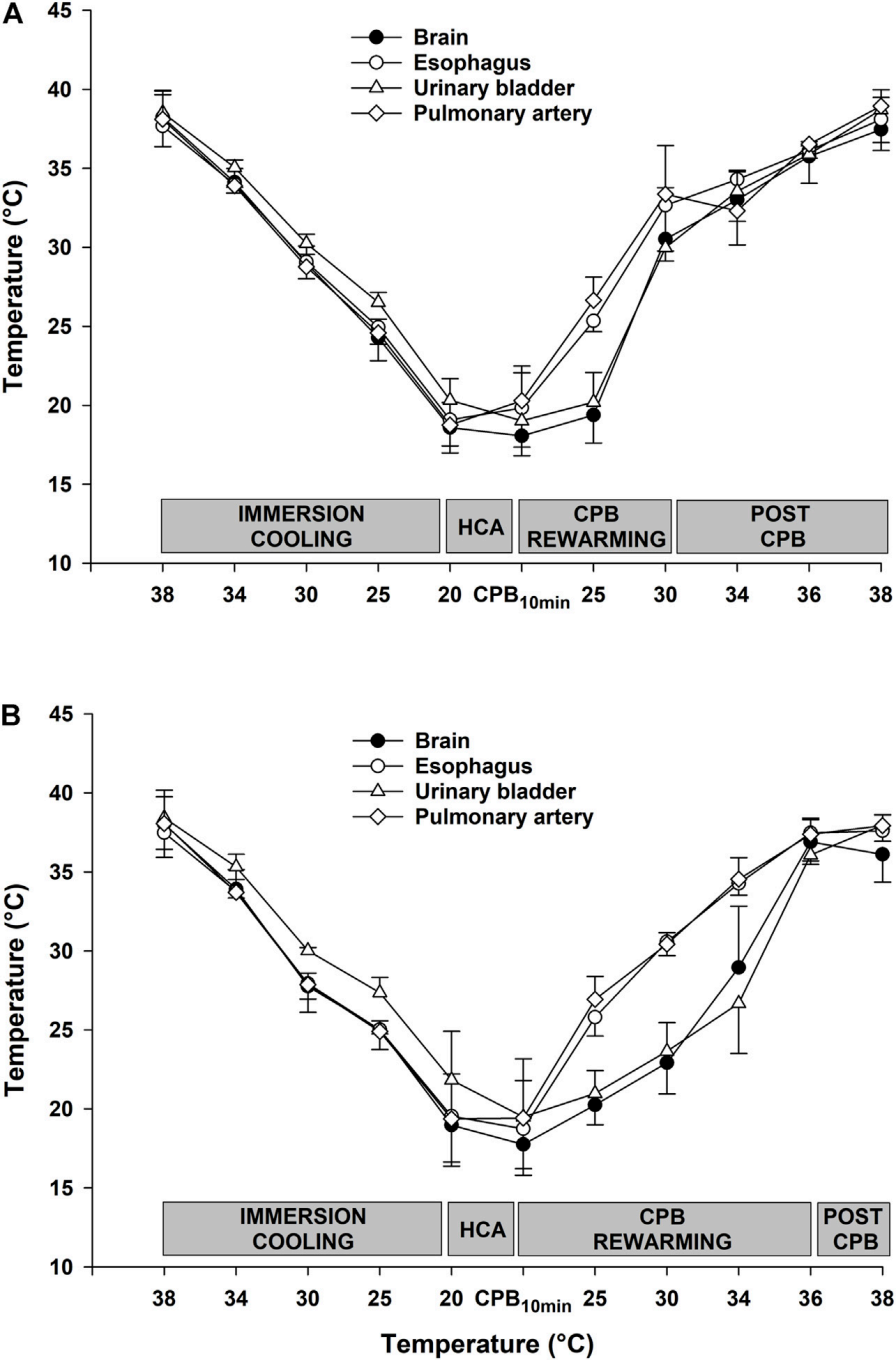
Temperatures at various body locations. **(A)**: CPB to 30°C and **(B)**: CPB to 36°C.

As shown in Figure 1, there were no differences between groups in time spent in cooling to 20°C, and time in HCA. However, the total time for rewarming was 60 min longer in the CPB_30_ group compared to the CPB_36_ group. The time for rewarming from 20 to 30°C on CPB was 65 min in both groups (i.e., a rewarming rate of 0.15°C/min or 9°C/h). In the CPB_36_ group, the time for rewarming from 30 to 36°C on CPB was 50 min (i.e., a rewarming rate of 0.12°C/min or 7°C/h. In contrast, the time for rewarming from 30 to 36°C by immersion in the CPB_30_ group was 110 min (i.e., a rewarming rate of 0.05°C/min or 3°C/h. The temperatures in different organs (esophagus, urinary bladder, pulmonary artery, and brain) during rewarming from 30° to 36°C on CPB in the CPB_30_ group were relatively consistent (Figure 2A) In contrast, the increase in temperature in the urinary bladder and brain was slower compared to other organs during rewarming from 30° to 36°C by immersion in the CPB_36_ group (Figure 2B).

### Survival

All animals in the CPB_36_ group survived, whereas three out of eight animals in the CPB_30_ group died from circulatory shock during weaning from CPB or during surface rewarming before reaching 36°C. However, this differences in survival between groups was not statistical different (*p* = 0.082). Results from non-surviving animals were excluded.

### Hemodynamic function, pressures, O_2_ variables, and cerebral blood flow

#### Immersion cooling

**FIGURE 3:**
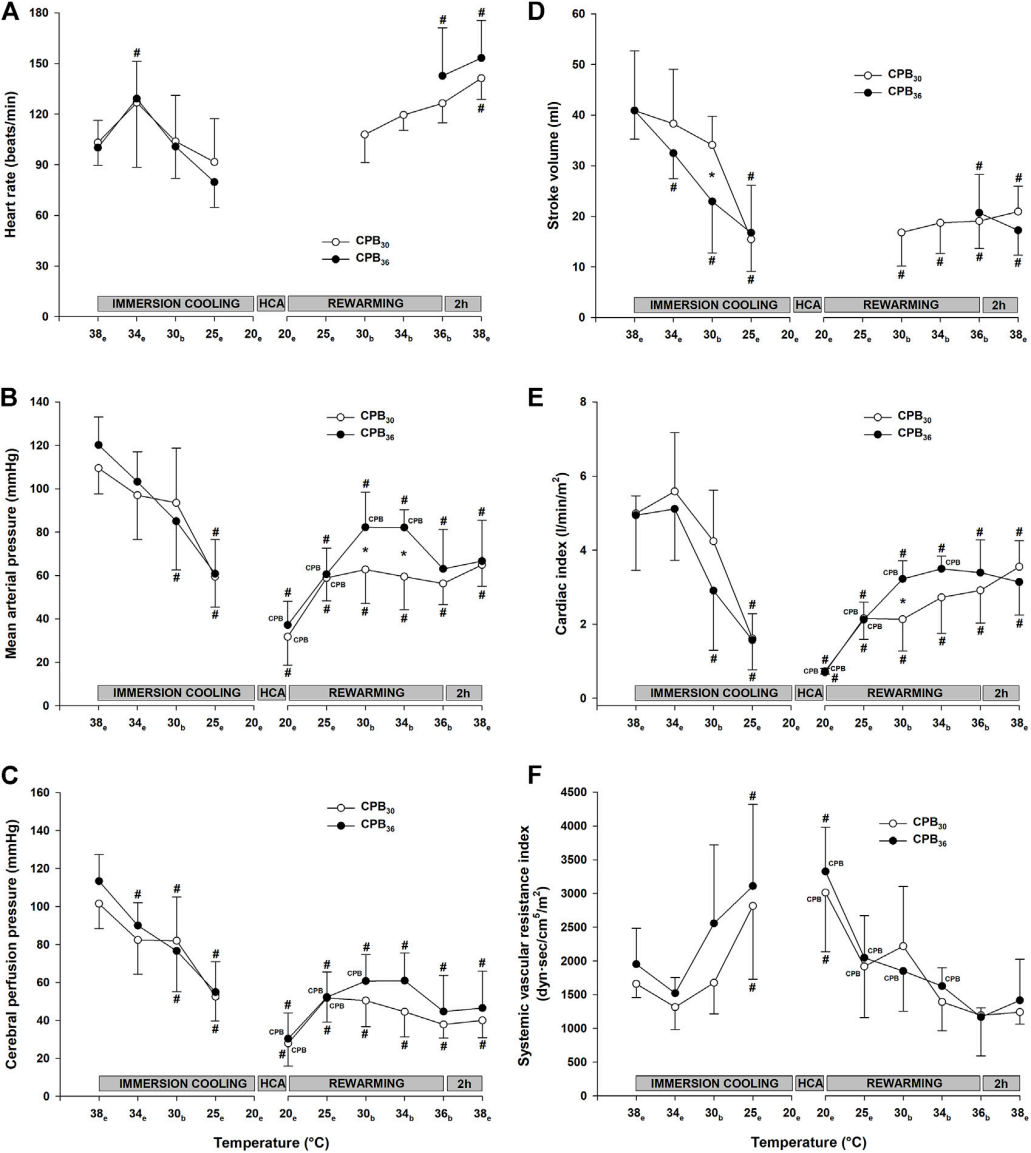
Hemodynamic variables. **(A)**: heart rate, **(B)**: mean arterial pressure, **(C)**: cerebral perfusion pressure, **(D)**: stroke volume, **(E)**: cardiac index, **(F)**: systemic vascular resistance. Temperature measured in: e – esophagus; b – bladder. Data presented as mean and SD. ^#^ *p* < 0.05 vs. intragroup baseline; *p < 0.05 vs. corresponding value in another group; ^**CPB**^ – variables measured during ongoing CPB.

During immersion cooling with spontaneous circulation a linear reduction in most hemodynamic functional variables was recorded before spontaneous cardiac arrest took place between 20 and 19°C (Figure 3). An exception was HR (Figure 3A), which increased significantly over the prehypothermic baseline value between 34° and 30°C but fell to below baseline during further cooling. Compared to baseline, mean arterial pressure (MAP), cerebral perfusion pressure (CPP), stroke volume (SV) and cardiac index (CI) at 25°C were all significantly reduced (Figures 3B–E). Differences between groups in systemic vascular resistance index (SVRI) appeared at 25°C; when compared to baseline resistance in the CBP_36_ group it was increased but remained unchanged in the CPB_30_ group (Figure 3F).

**FIGURE 4:**
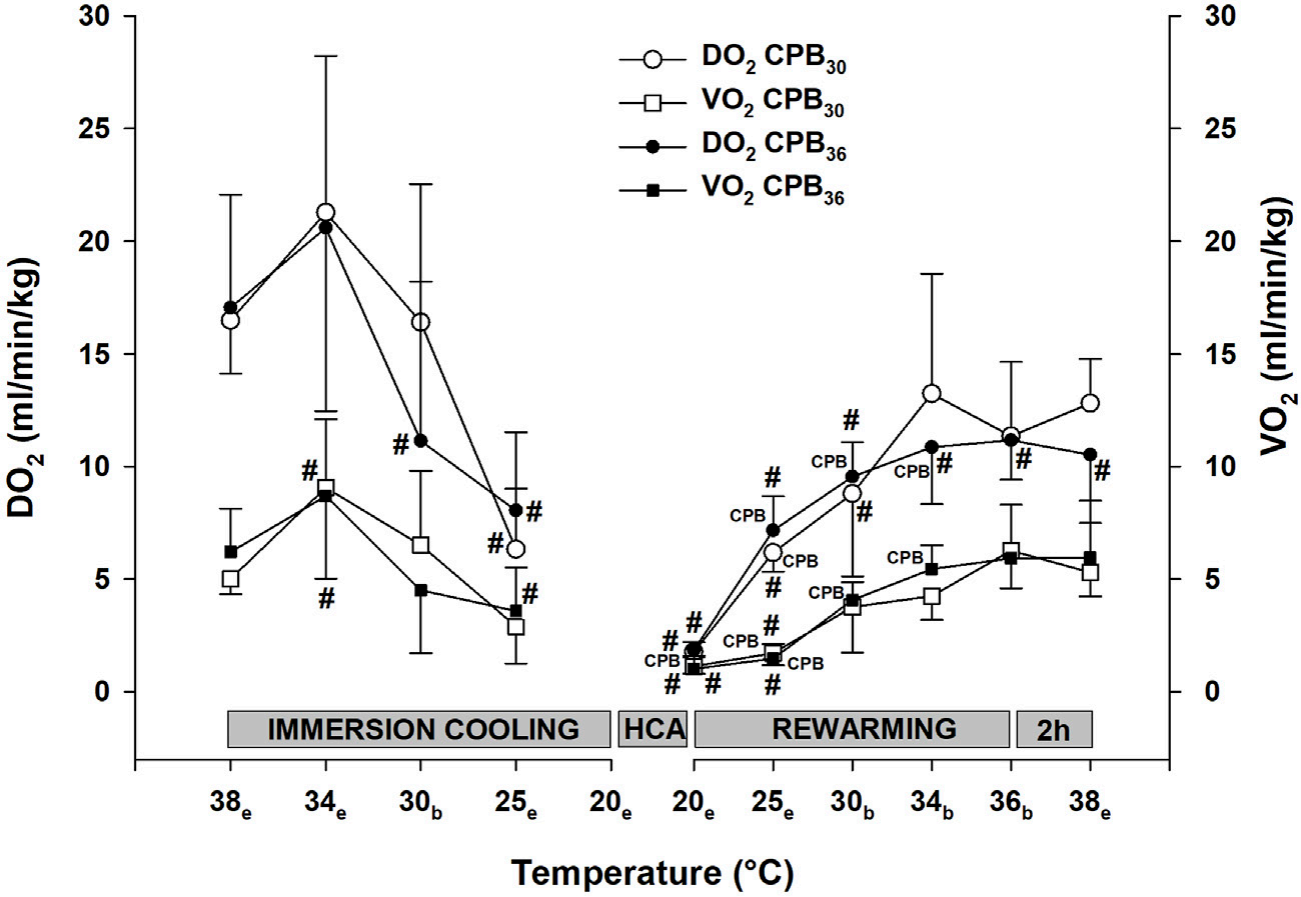
Oxygen transport. DO_2_ – Global oxygen delivery, VO_2_ – oxygen uptake. Temperature measured in: e – esophagus; b – bladder. Data presented as mean and SD. ^#^ *p* < 0.05 vs. intragroup baseline; ^**CPB**^ – variables measured during ongoing CPB.

**FIGURE 5:**
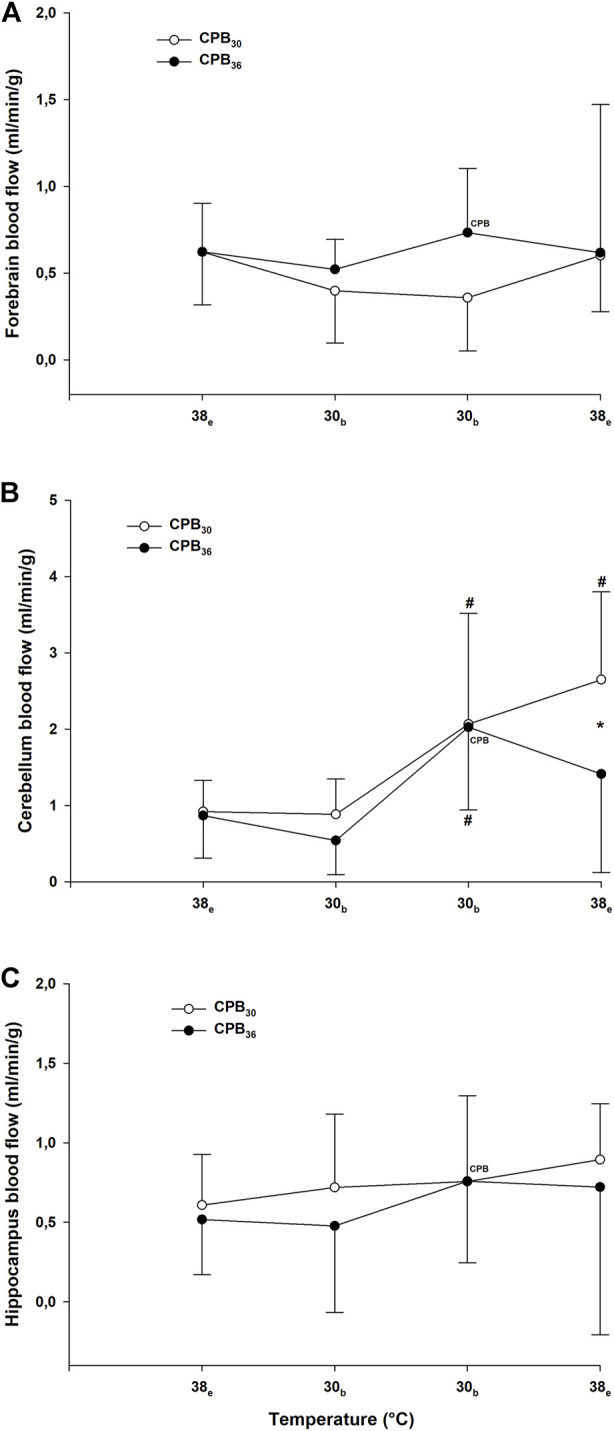
Cerebral blood flow. **(A)**: forebrain; **(B)**: cerebellum; **(C)**: hippocampus. Temperature measured in: e – esophagus; b – bladder. Data presented as mean and SD. ^#^ *p* < 0.05 vs. intragroup baseline; *p < 0.05 vs corresponding value in another group; ^**CPB**^ – variables measured during ongoing CPB.

Parallel to the reduction in CI, global DO_2_ was significantly reduced at 25°C in both groups, whereas VO_2_ remained unchanged (Figure 4). Cooling to 30°C did not cause any change in cerebral blood flow (forebrain, cerebellum, and hippocampus) when compared to baseline (Figure 5).

#### Rewarming on cardio-pulmonary bypass to 30°C

As per protocol, circulatory function during CPB rewarming was determined as the pump flow necessary to maintain a MAP > 50 mmHg and a central venous O_2_ saturation > 60%. These requirements were met, but when compared to prehypothermic baseline values, both MAP (Figure 3B) and CI (Figure 3E) were significantly lower in both groups, whereas SVRI (Figure 3F) returned to baseline. CPP was significantly reduced below baseline in both groups (Figure 3C). During CPB at 20°C, before rewarming started, both DO_2_ and VO_2_ (Figure 4) were close to zero, but increased during rewarming at 25°C. At 30°C VO_2_ had returned to baseline in both groups, whereas DO_2_ remained reduced. At 30°C, both groups showed a significant increase in blood flow in the cerebellum over that measured at baseline (Figure 5B). Blood flow in forebrain and hippocampus remained unaltered throughout the experimental protocol (Figures 5A,C).

#### Rewarming on cardio-pulmonary bypass to 36°C

Both MAP (Figure 3C) and CI (Figure 3E) remained significantly below baseline during rewarming, whereas SVRI returned to baseline in (Figure 3F). At 30°C, VO_2_ had returned to baseline, but DO_2_ remained reduced (Figure 4). Cerebellar blood flow (Figure 5B) increased above baseline level at 34°C, despite a significant reduction in CPP (Figure 3C).

#### Immersion rewarming to 36°C

During spontaneous circulation after weaning from CPB at 30°C, both MAP (Figure 3C) and CI (Figure 3E) remained significantly below baseline, whereas SVRI returned to baseline (Figure 3F). At 30°C, VO_2_ returned to baseline, DO_2_ increased but returned to baseline first during rewarming from 34°C (Figure 4). Cerebellar blood flow (Figure 5B) increased above baseline level at 34°C, despite a significant reduction in CPP (Figure 3C).

Of the three non-surviving animals in this group, two of them had electromechanical dissociation during attempts to wean from CPB at 30°C. In the remaining animal CI increased during rewarming to 34°C (2.4 l/min) but showed a progressive fall in CI during rewarming to 36°C (1.5 l/min) and the animal did not survive the ensuing 2 h.

#### Immersion rewarming 36–38°C

During spontaneous circulation in both groups, SV remained significantly reduced compared to baseline (Figure 3D). The significant increase in HR (Figure 3A) in both groups was most likely due to the pharmacologic effects of dopamine. CI (Figure 3E) remained significantly reduced except for at the end of rewarming (38°C) in the CPB_30_ group, where CI returned to baseline in parallel with the increase in HR. At 38°C the significant increase in blood flow in the cerebellum above baseline remained only in the CPB_30_ (Figure 5B).

### Brain metabolism

**FIGURE 6:**
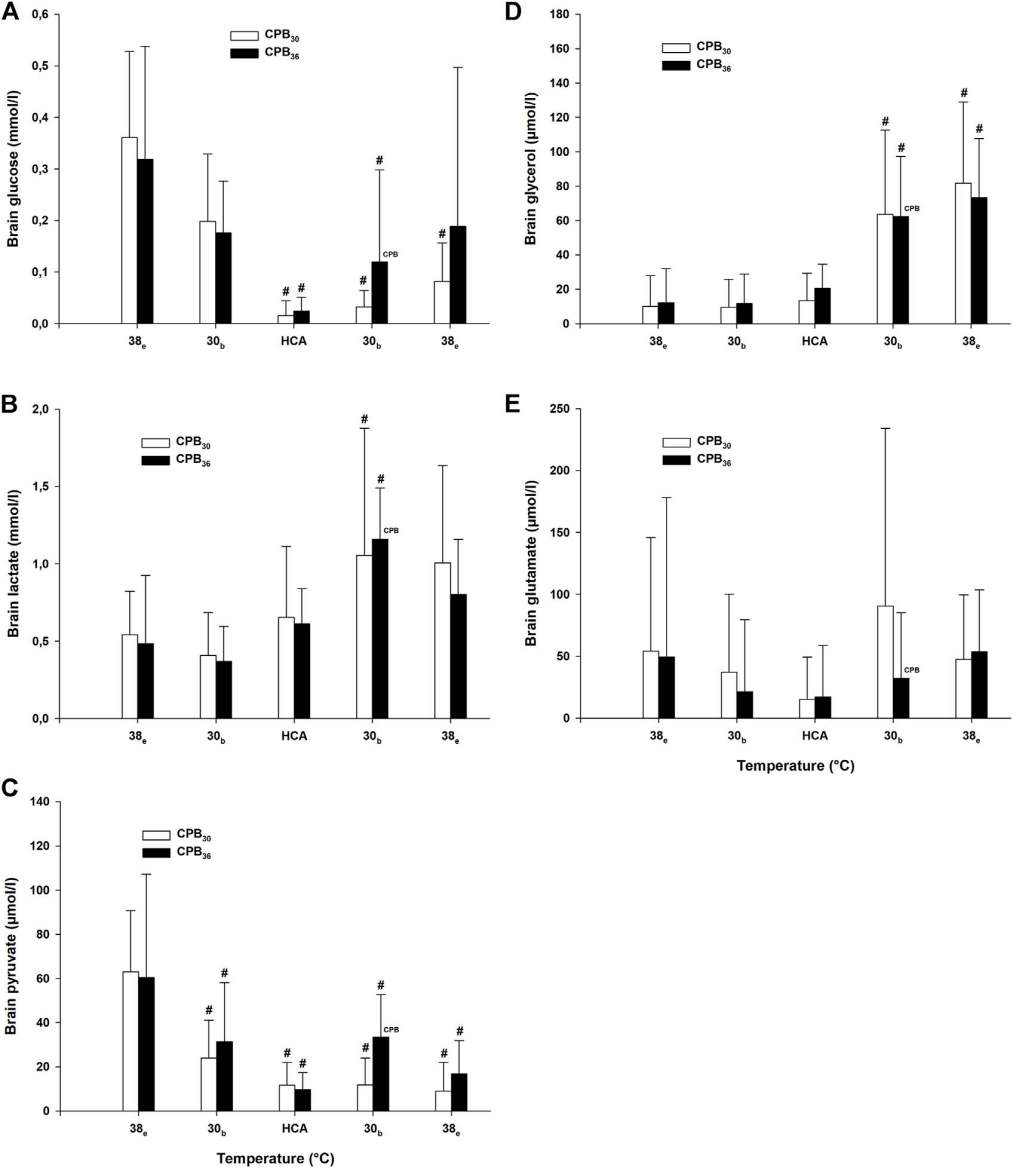
Brain microdialysate analyses. **(A)**: brain glucose, **(B)**: brain lactate, **(C)**: brain pyruvate, **(D)** brain glycerol, **(E)**: brain glutamate. Temperature measured in: e – esophagus; b – bladder. HCA – hypothermic cardiac arrest. Data presented as mean and SD. ^#^ *p* < 0.05 vs. intragroup baseline; ^**CPB**^ – variables measured during ongoing CPB.

**FIGURE 7:**
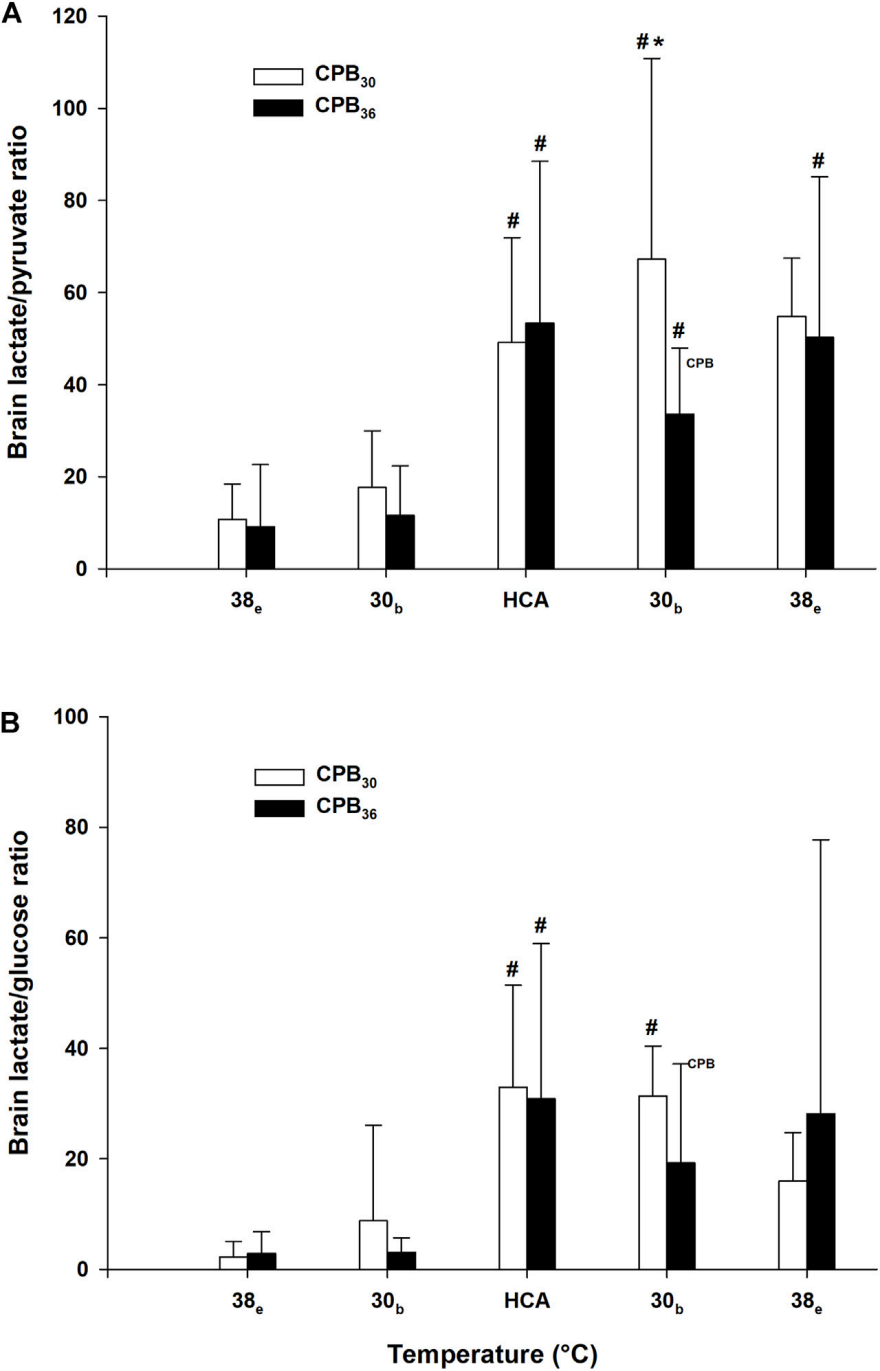
Brain microdialysate analyses (cont.). **(A)**: brain lactate/pyruvate ratio, **(B)**: brain lactate/glucose ratio. Temperature measured in: e – esophagus; b – bladder. Data presented as mean and SD. ^#^ *p* < 0.05 vs. intragroup baseline; *p < 0.05 vs. corresponding value in another group; ^**CPB**^ – variables measured during ongoing CPB.

Brain glucose was significantly reduced in both groups during HCA arrest and during rewarming at 30°C (Figure 6A). After rewarming to 38°C, glucose was reduced in the CPB_30_ group but returned to baseline in the CPB_36_ group. Lactate levels were increased in both groups after rewarming to 30°C (Figure 6B). Pyruvate increased significantly during cooling in both groups at 30°C, remained elevated throughout the rest of the experimental protocol but returned to baseline after final rewarming to 38°C (Figure 6C). Glycerol levels increased in both groups after rewarming to 30°C and remained elevated throughout the rest of the experiment (Figure 6D). No significant changes in glutamate levels were seen during cooling/rewarming (Figure 6E). The lactate/pyruvate ratio was significantly increased during HCA and rewarming from 30–38°C, except for a return to baseline in the CPB_30_ group at 38°C (Figure 7A). The lactate/glucose ratio was increased during HCA, remained elevated in the CPB_30_ group during rewarming at 30°C, and returned to within baseline levels after final rewarming to 38°C (Figure 7B).

### Biochemical variables

Plasma albumin values were significantly reduced below prehypothermic baseline during both CPB and immersion rewarming to 38°C (Table 1). After rewarming to 38°C, plasma levels of ALAT, ASAT, and cTnT were significantly increased above corresponding levels measured at baseline and during CPB at 20°C in both groups.

**TABLE 1:** Blood plasma albumin, ASAT, ALAT and cTnT values.

	Experimental group	38°C-BL	20°C-10 min CPB	38°C-2 h post RW
Albumin (g/L)	CPB_**30**_	30 ± 7	19 ± 6^#^	22 ± 3^#^
	CPB_**36**_	34 ± 2	18 ± 3^#^	23 ± 4^#^
ASAT (U/L)	CPB_**30**_	44 ± 20	85 ± 51	397 ± 272^#†^
	CPB_**36**_	43 ± 21	67 ± 18	339 ± 152^#†^
ALAT (U/L)	CPB_**30**_	62 ± 15	47 ± 18	106 ± 24^#†^
	CPB_**36**_	70 ± 16	42 ± 7^#^	91 ± 20^†^
cTnT (ug/L)	CPB_**30**_	0.03 ± 0.02	0.11 ± 0.09	4.2 ± 3.2^#†^
	CPB_**36**_	0.02 ± 0.03	0.1 ± 0.05	3.6 ± 2.3^#†^

ASAT – aspartate transaminase, ALAT – alanine transaminase, cTnT – cardiac troponin T. Data presented as mean and SD. ^#^p<0.05 vs. intragroup 38°C-BL, ^†^p<0.05 vs. intragroup 20°C-10 min CPB.

After the start of CPB rewarming at 20°C, hematologic counts; white cells, red cells, blood platelets, and hemoglobin were all significantly reduced, as was also hematocrit (Table 2). The only exception was plasma glucose level, which remained unaltered throughout the experiment. After final rewarming to 38°C, except for white blood cell count, which was significantly elevated above baseline, all other hematologic variables as well as hematocrit returned to within their respective baseline levels.

**TABLE 2:** Hematology and blood glucose.

	Experimental group	38°C-BL	20°C-10 min CPB	38°C-2 h post RW
Red blood cell count (10^12^/L)	CPB_**30**_	5 ± 0.3	3.9 ± 0.4^#^	5.2 ± 0.5^†^
	CPB_**36**_	5.2 ± 0.4	4.2 ± 0.2^#^	5.1 ± 0.4^†^
White blood cell count (10^6^/L)	CPB_**30**_	20.3 ± 2.8	4.3 ± 1^#^	29.9 ± 10^#†^
	CPB_**36**_	21.9 ± 4.7	5.6 ± 2.7^#^	26.7 ± 14^†^
Blood platelet count (10^9^/L)	CPB_**30**_	713 ± 186	312 ± 120^#^	723 ± 209^†^
	CPB_**36**_	624 ± 121	249 ± 70^#^	549 ± 132^†^
Hemoglobin (g/dl)	CPB_**30**_	8.7 ± 0.7	6.4 ± 0.8^#^	9.3 ± 1.1^†^
	CPB_**36**_	8.6 ± 0.8	6.8 ± 0.4^#^	8.6 ± 1^†^
Hematocrit (%)	CPB_**30**_	27.3 ± 2.6	20.6 ± 3^#^	27.4 ± 4.3^†^
	CPB_**36**_	28.5 ± 3.1	22.4 ± 1.5^#^	26.9 ± 2.5^†^
Glucose (mmol/L)	CPB_**30**_	5.1 ± 1.1	7.2 ± 3.4	6.3 ± 1.1
	CPB_**36**_	3.8 ± 1.3	5.6 ± 3.3	5 ± 1.6

Data presented as mean and SD. #p<0.05 vs. intragroup 38°C-BL, †p<0.05 vs. intragroup 20°C-10 min CPB.

Table 3 shows changes in blood gas variables and electrolytes during the experiment. Despite significant changes, most were moderate, or they operated within physiologic levels. Of note is the significant increase in potassium above baseline in both groups measured after start of CPB at 20°C. This elevation in potassium vanished after rewarming to 38°C in CPB_30_ animals but remained in the CPB_36_ group.

**TABLE 3:** Arterial blood gases values.

	Experimental group	38°C-BL	20°C-10 min CPB	38°C-2 h post RW
pH	CPB_**30**_	7.55 ± 0.05	7.3 ± 0.12^#^	7.39 ± 0.07^#^
	CPB_**36**_	7.54 ± 0.08	7.31 ± 0.13^#^	7.37 ± 0.08^#^
pCO_2_ (kPa)	CPB_**30**_	5.1 ± 0.7	6.5 ± 2.3	5.5 ± 1.6
	CPB_**36**_	5.3 ± 1.1	6.7 ± 1.6	5.7 ± 0.8
pO_2_ (kPa)	CPB_**30**_	25.3 ± 7.3	28.4 ± 2.2	29.6 ± 16
	CPB_**36**_	22.7 ± 7.7	25 ± 8.1	26.4 ± 14
Sat (%)	CPB_**30**_	99.1 ± 1.1	99.6 ± 0.7	99.1 ± 0.8
	CPB_**36**_	99.4 ± 0.9	99.5 ± 0.5	96.3 ± 5.3
BE (mmol/L)	CPB_**30**_	9.5 ± 3.1	−4.2 ± 3.3^#^	−0.2 ± 3.1^#†^
	CPB_**36**_	10.1 ± 1.9	−2 ± 3.2^#^	−0.1 ± 4.6^#^
Na^+^ (mmol/L)	CPB_**30**_	138 ± 7	138 ± 7	144 ± 10^#^
	CPB_**36**_	137 ± 4	136 ± 2	138 ± 4
K^+^ (mmol/L)	CPB_**30**_	3.5 ± 0.3	7.6 ± 1.0^#^	4.3 ± 0.5^†^
	CPB_**36**_	3.6 ± 0.4	8.8 ± 1.3^#*^	4.4 ± 0.6^#†^

pCO_2_ - venous partial O_2_ pressure, pO_2_ - arterial partial O_2_ pressure, Sat - arterial O_2_ saturation, BE - base excess. Data presented as mean and SD. #p<0.05 vs. intragroup 38°CBL, †p<0.05 vs. intragroup 20°C-10 min CPB, *p<0.05 vs. corresponding value in the CPB30 group.

### Fluid administration, weight gain ratio, and organ dry/wet wt. ratio

**FIGURE 8:**
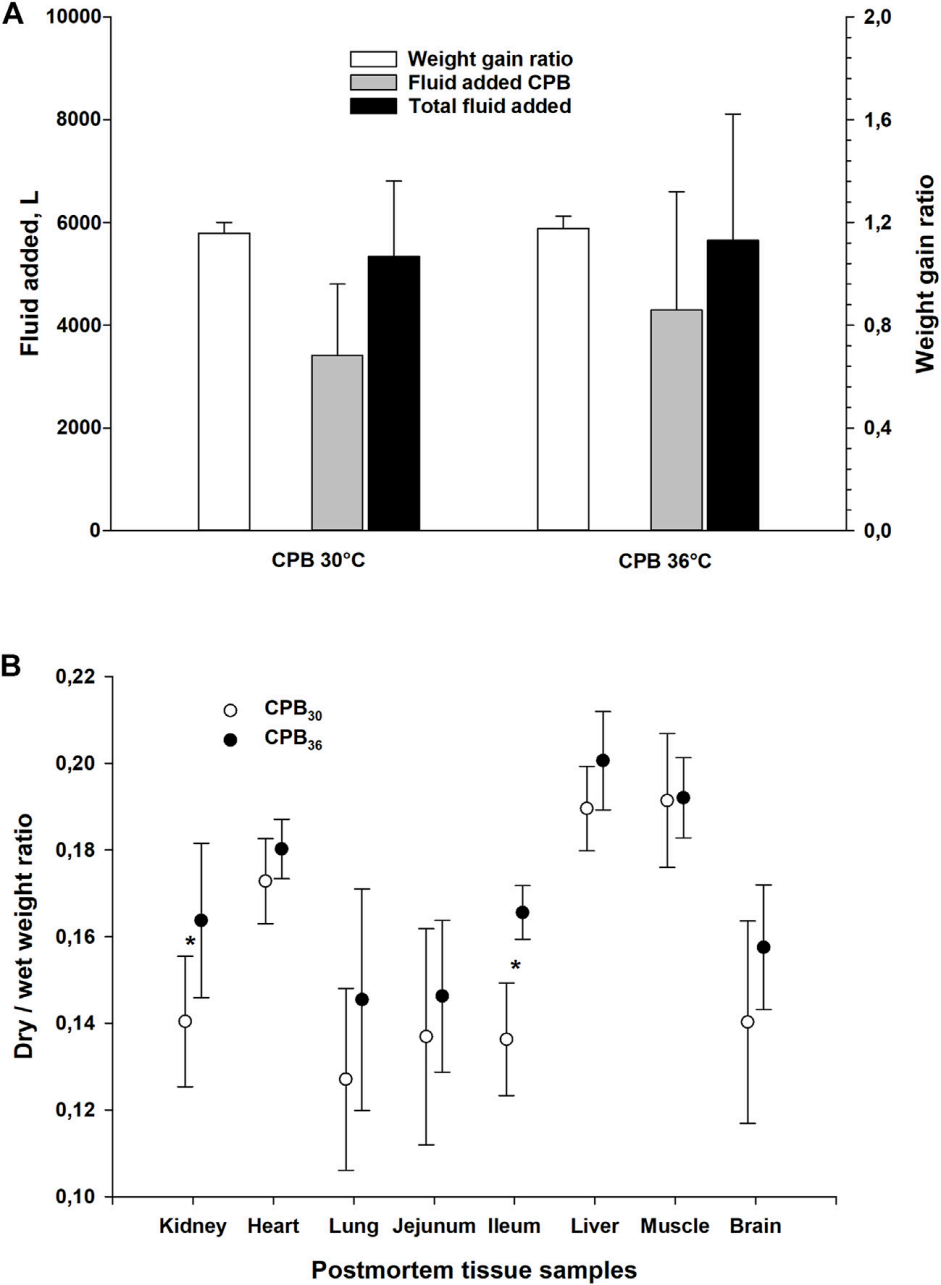
Weight gain ratio and fluid added, and post-mortem dry to wet weight ratio in organs tissue samples. **(A)**: weight gain ratio and fluid added during experiment (on CPB and total), **(B)**: post-mortem dry to wet weight ratio in different organs. Data presented as mean and SD. *p < 0.05 vs. corresponding value in another group.

There was no difference between groups in fluid volume administration during CPB rewarming if weaning took place at 30 or 36°C (Figure 8A). Further, no difference between groups in total fluid volume given during experiment was seen after rewarming to 38°C. Likewise, the increase in body weight, was not different between groups. Post-mortem organ dry/wet weight ratios indicated a statistically significant increase in water content in the kidney and the gut in the CPB_30_ group compared to the CPB_36_ group (Figure 8B).

In both groups, CVP and ICP were significantly increased after start of CPB rewarming at 20°C (Table 4). After final rewarming to 38°C, these values increased above those measured at 20°C.

**TABLE 4:** CVP and ICP values.

	Experimental group	38°C-BL	20°C-10 min CPB	38°C-2 h post RW
CVP (mmHg)	CPB_**30**_	7 ± 4	8 ± 6	10 ± 4^#†^
	CPB_**36**_	8 ± 3	8 ± 4	15 ± 2^#†^
ICP (mmHg)	CPB_**30**_	8 ± 3	8 ± 4	25 ± 10^#†^
	CPB_**36**_	7 ± 3	8 ± 4	20 ± 5^#†^

CVP – central venous pressure, ICP – intracranial pressure. Data presented as mean and SD. ^#^p<0.05 vs. intragroup 38°C-BL, ^†^p<0.05 vs. intragroup 20°C-10 min CPB.

## Discussion

The results of this study indicate that following 75 min of HCA at 20°C, despite using different rewarming rates and early vs. late weaning from CPB, all surviving animals displayed significant acute cardiac dysfunction after rewarming. Further, as the need for vascular volume supply was greatest during CPB rewarming < 30°C, differences between groups in CPB exposure time, or rewarming rate > 30°C, did not create differences in total need for vascular volume supply neither during CPB nor during immersion rewarming to 38°C. Also, the different rewarming strategies created no differences between groups in cerebral metabolic variables, or cerebral pressures and blood flow.

### Cardiac function

In the pioneering experiments exploring the potential role of hypothermia in cardiac surgery, a significant reduction in CO after rewarming experimental animals was reported (Bigelow et al., 1950). This initial observation was confirmed by subsequent findings in experiments in the intact animal heart, cooled in a non-arrested state, and document the existence of hypothermia-induced cardiac dysfunction and reduced CO after rewarming (Ross, 1954; Blair et al., 1956; Fedor et al., 1958; Popovic and Kent, 1965; Steen et al., 1980; Tveita et al., 1996a). However, the introduction of CPB in cardiac surgery (Sealy et al., 1957) appeared to eliminate the occurrence of post-hypothermic cardiac dysfunction. Subsequently, post-operative cardiac dysfunction has been attributed to the preceding period of ischemia-reperfusion during cardiac surgery and not to the hypothermic exposure (Toller and Metzler, 2005). Therefore, post-hypothermic cardiac dysfunction remains a clinical challenge related only to rewarming from accidental hypothermia.

During hypothermia excitation-contraction coupling of cardiac muscle force generation is altered (Han et al., 2010; Schaible et al., 2016; Tveita et al., 2019), and Ca^2+^ overload may occur (Tani and Neely, 1989; Vassalle and Lin, 2004), both mechanisms that may contribute to cardiac dysfunction.

However, in isolated cardiac myocytes the amplitude and duration of the evoked [Ca^2+^]_cyt_ transient returns to normothermic levels during rewarming (Schaible et al., 2016). Thus, it does not appear that the decrease in contractility of cardiac myocytes (heart failure) after rewarming can be solely attributed to dysregulation of [Ca^2+^]_cyt_ release and reuptake. We have documented, by use of isolated, electrically stimulated myocytes cooled to 15°C and rewarmed, that the reduction in force generation, independent of [Ca^2+^]_cyt_ levels, is the effect of an increased phosphorylation of cardiac troponin I (cTnI) leading to reduced Ca^2+^ sensitivity of the contractile response after rewarming (Han et al., 2010; Schaible et al., 2016; Schaible et al., 2018; Tveita et al., 2019).

### Extravasation of fluid

Extravasation of plasma fluid takes place during normothermic CPB as well as during cooling with intact circulation (Tveita et al., 1996b; Hammersborg et al., 2005), but increases significantly during hypothermic CPB (Heltne et al., 2001; Farstad et al., 2003; Farstad et al., 2005; Hammersborg et al., 2005). In a recent animal experiment (Filseth et al., 2022), we reported that significantly more fluid volume was needed to achieve adequate perfusion during CPB rewarming from accidental hypothermia over that required in animals after hypothermic cardiac surgery. The results of the present study demonstrated that the need for vascular volume replacement during CPB rewarming was similar between groups, irrespective if terminated during rewarming at 30°C or at 36°C. This indicates that capillary leak during CPB was greatest below 30°C.

### Neuroprotection

The potential of hypothermia to limit ischemia-reperfusion injuries and to provide cardio- and neuroprotection is well established. In addition to reducing metabolic rate and O_2_ consumption, neuroprotective effects of therapeutic hypothermia during as well as after ischemia/reperfusion is ascribed to a reduction in the production of reactive O_2_ species (ROS) during regional and global reperfusion of the brain (Rosomoff and Holaday, 1954; Rout et al., 2020). In general, for most organs there is no definite association between the presence of tissue edema and the degree of reduced tissue function, nor organ survival. One exception is the brain, where tissue edema and elevated ICP may quickly lead to cessation of cerebral circulation.

In the present study, we monitored ICP, and measured cerebral blood flow and metabolism, in addition to global circulatory function, but found no decrease in blood flow during rewarming even though CPP was reduced. Despite using rewarming strategies aimed at reducing tissue edema, ICP increased significantly. Furthermore, brain lactate levels were significantly increased in both groups after 75 min in HCA and CPB rewarming to 30°C, but lactate and cerebral blood flow returned to baseline levels after rewarming to 38°C. However, the simultaneous reduction in brain glucose and pyruvate levels, the rise in lactate to pyruvate ratio, and the increased glycerol levels, indicate an alteration of cerebral metabolism during HCA, as well as during rewarming to 30°C. Altogether, these results indicate a good neurological outcome, despite prolonged periods of no-flow or low-flow, especially as hypothermia was established before cardiac arrest (1986; Walpoth et al., 1990; van den Broek et al., 2010; Paal et al., 2016).

### Cardio-pulmonary bypass—side effects

Although the overall morbidity and mortality associated with use of CPB has steadily decreased since its introduction in cardiac surgery (Sealy et al., 1958), central nervous system complications and cardiovascular dysfunction continue to account for significant rates of morbidity and mortality (Newman et al., 1995; Grigore et al., 2002; Grigore et al., 2009; Bartels et al., 2013; Debaty et al., 2016). The central nervous system complications range from subtle cognitive, personality, and behavior changes to catastrophic stroke and death (Selnes et al., 1999; Newman et al., 2006), in addition to a high incidence of severe cardiovascular dysfunction after rewarming (Salerno et al., 1991; Borger et al., 2001; Nussmeier, 2002). These complications can be partly explained as a result of ischemia-reperfusion injury, but they can also be aggravated by the rewarming strategies used as well as by the existence of hypothermia-induced cardiac dysfunction. Therefore, it may be possible to further reduce overall morbidity when using CPB in cardiac surgery, as well as in rewarming from accidental hypothermia, by adjusting the rate of rewarming and the rewarming strategy used.

### Cardio-pulmonary bypass—rate of rewarming

Numerous studies have promoted hypothermic CPB in attenuating the central nervous system side effects of cardiac surgery, whereas other studies have clearly demonstrated that hyperthermia in the perioperative period create harmful effects on neurocognitive function (Mora et al., 1996; Grigore et al., 2009). It has been postulated that an overshoot of cerebral temperature can occur when larger temperature gradients are required to create a more rapid CPB rewarming. Thus, in this debate, the rate of rewarming has become an issue. A study on pigs reported improved survival outcomes after slow rewarming (0.5°C/min) vs. fast rewarming (1°C/min) from profound hypothermia (Alam et al., 2006). A prospective, randomized, clinical study compared neurocognitive performance after CPB rewarming to 37°C vs. limited CPB rewarming to 33°C, followed by passive rewarming to 37°C, and found significantly better performance in the latter patient group (Sahu et al., 2009). These studies clearly indicated a positive effect on neurocognitive function by avoiding hyperthermia, but the ideal rewarming rate remains unknown and requires further investigation (Saczkowski et al., 2018).

Rewarming rates reported in accidental hypothermia case series range from 1.5 to 10°C/h, and as in cardiac surgery avoiding hyperthermia appears to be essential for optimal neurocognitive outcome, although optimal rewarming rate remains unknown (Grigore et al., 2009). Weaning from CPB rewarming at core temperatures > 31°C is recommended (Paal et al., 2016), and a slower CPB rewarming rate (≤ 5.0°C/h) in severe accidental hypothermia was associated with improved survival and good neurological outcomes (Saczkowski et al., 2021).

In the present study, the rewarming strategies were based on a compromise between i) Minimizing CPB exposure time at temperatures < 30°C to reduce excessive transcapillary fluid loss; and ii) Achieving a slower rewarming rate (< 5.0°C/h) during rewarming from 30 to 36°C. Thus, CPB rewarming rate from 20 to 30°C was 0.15°C/min (9°C/h) in both groups, whereas rewarming rate from 30 to 36°C was 0.05°C/min (3°C/h) using immersion, and 0.12°C/min (7°C/h) using CPB.

### Cardio-pulmonary bypass—its application as rewarming method in accidental hypothermia

Survival rates of accidental hypothermia patients without neurologic impairment after CPB rewarming ranges from 47 to 63% in different studies (Walpoth et al., 1997; Farstad et al., 2001; Silfvast and Pettila, 2003; Ruttmann et al., 2007). Thus, improved rewarming strategies for accidental patients may result in reducing mortality rate (Saczkowski et al., 2018). Unfortunately, in a survey from a Norwegian university hospital, no change in survival rate was reported over the past 30 years when using CPB to rewarm accidental hypothermia patients in HCA (Svendsen et al., 2017).

The methods used to rewarm accidental hypothermia patients have been mostly adapted from cardiac surgery where CPB has been routinely applied to cool and rewarm patients. The extensive volume of cardiovascular research, both clinical and preclinical, conducted over the past 60 years has been a prerequisite for safe use of CPB in cardiac surgery. In contrast, there is a lack of documentation for best use of CPB to rewarm accidental hypothermia patients. Information regarding the use of CPB in cardiac surgery appears to be not directly applicable for rewarming accidental hypothermia patients.

### Alternative method to cardio-pulmonary bypass rewarming

Extracorporal membrane oxygenation (ECMO) has been increasingly used to rewarm accidental hypothermia patients in HCA or with an unstable perfusing rhythm. The advantages of ECMO include the fact that it is rapidly available, requires less anticoagulation, and circulatory/respiratory support can be continued for days after rewarming if needed. An Austrian study showed a 6-fold increase in survival in accidental hypothermia patients rewarmed with ECMO vs. CPB (Ruttmann et al., 2007). Similarly, a Japanese study reported a marked increase in survival of accidental hypothermia patients (from 46 to 84%) with the use of ECMO rewarming (Morita et al., 2011), which now appears to be state of the art in rewarming accidental hypothermia patients in HCA.

### Limitations

It may be argued that attempts to wean from CPB in the CPB_30_ group should have been delayed until the core temperature had reached 31–34°C as recommended in clinical guidelines. However, our intention was to increase differences between groups with respect to duration of hypothermic CPB as much as possible. Weaning from CPB was technically feasible at 30°C, at least in six of the eight animals. If weaning had been done at higher temperatures this may have increased the number of animals rewarmed to 38°C but would not have led to changes in our main conclusions.

## Conclusion

Using a porcine experimental model of accidental HCA, the use of two different CPB rewarming strategies aimed at attaining organ protective effects were compared. As 3 out of 8 animals did not survive weaning from CPB at 30°C, early weaning gave no advantages over weaning at 36°C. Further, in surviving animals, the results showed no differences between groups in the need for vascular volume replacement, nor any differences in cerebral blood flow or pressures. However, the need for CPB rewarming to compensate post-hypothermic cardiac dysfunction was displayed. Clinical reports favor the use of ECMO rewarming in accidental hypothermia, but extensive research remains to customize the use of this technique to achieve optimal organ protection and increased survival in these patients.

## Data Availability

The raw data supporting the conclusions of this article will be made available by the authors, without undue reservation.

## Supplementary material

Supplementary Figure S1 Intraoperative image of the heart cannulation.
