# Supplementary figures and images for: Functional recovery after accidental deep hypothermic cardiac arrest: Comparison of different cardiopulmonary bypass rewarming strategies
Source: Front Physiol. 2022 Sep 5;13:960652. doi: 10.3389/fphys.2022.960652 (PMC9483155; doi:10.3389/fphys.2022.960652)

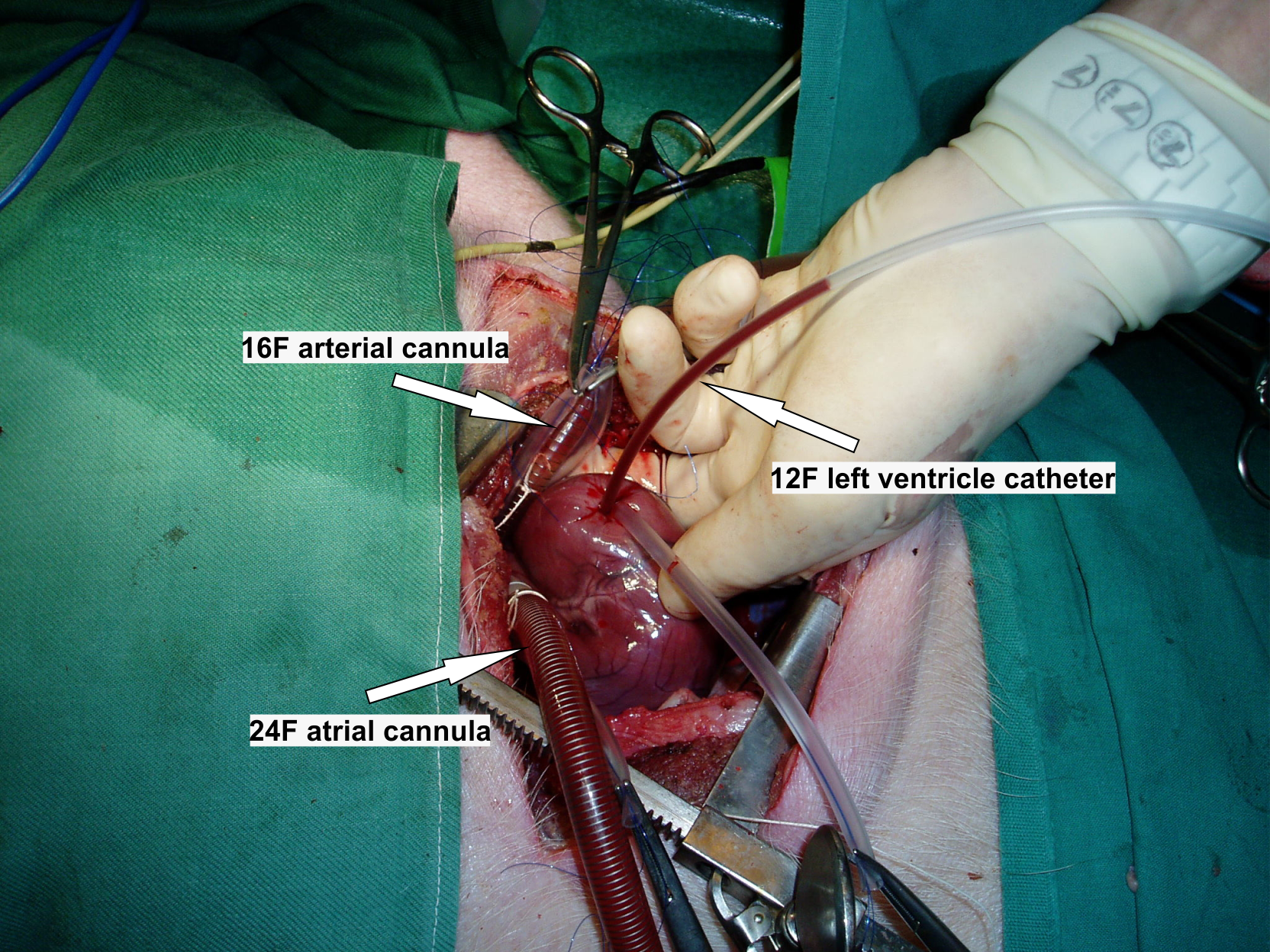

Supplement: Supplementary file 1 [file Image1.TIF]
